# Supplementary figures and images for: External mechanical loading overrules cell-cell mechanical communication in sprouting angiogenesis during early bone regeneration
Source: PLoS Comput Biol. 2023 Nov 13;19(11):e1011647. doi: 10.1371/journal.pcbi.1011647 (PMC10681321; doi:10.1371/journal.pcbi.1011647)

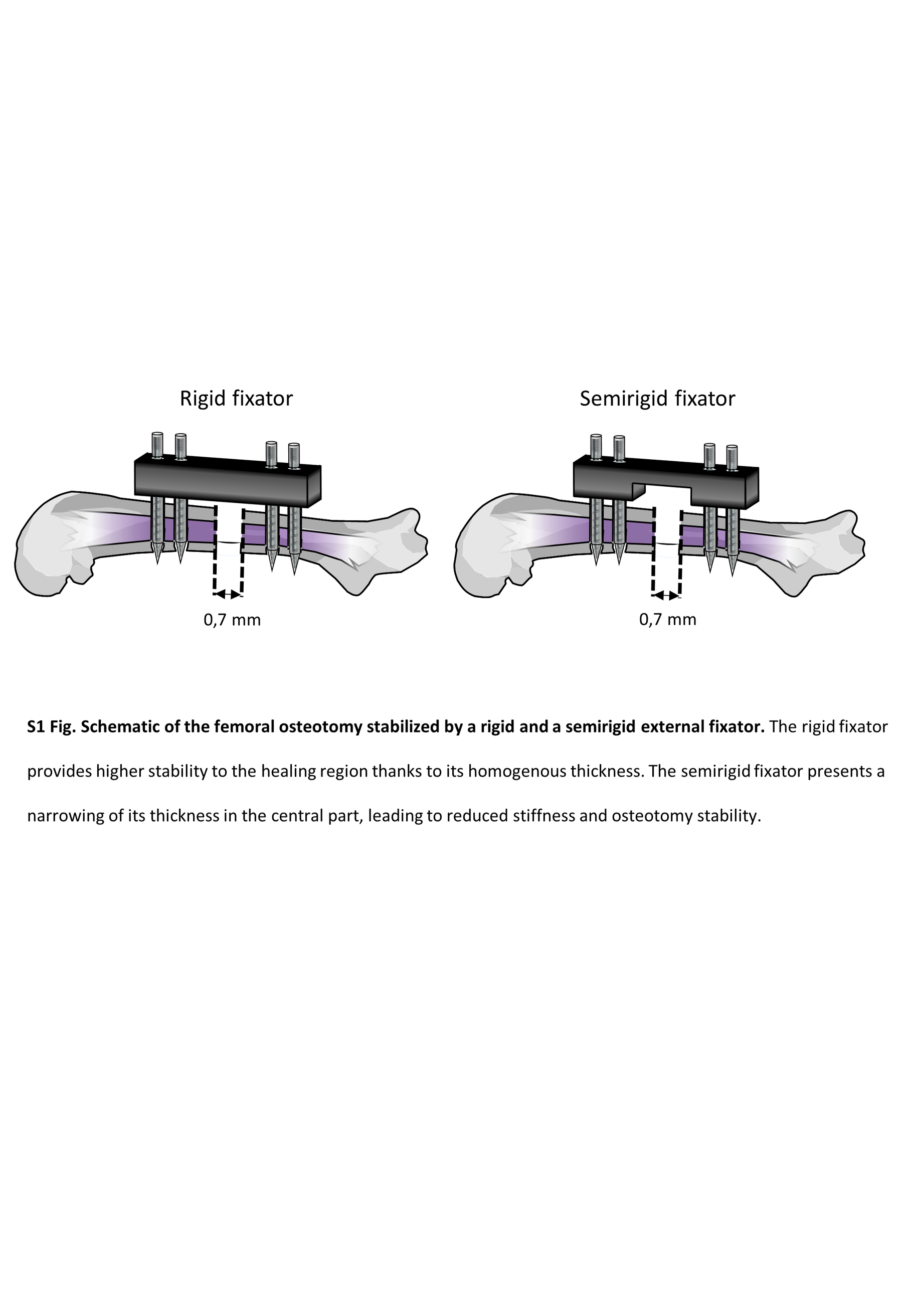

Supplement: S1 Fig — The rigid fixator provides higher stability to the healing region thanks to its homogenous thickness. The semirigid fixator presents a narrowing of its thickness in the central part, leading to reduced stiffness and osteotomy stability. (TIF) [file pcbi.1011647.s001.tif]

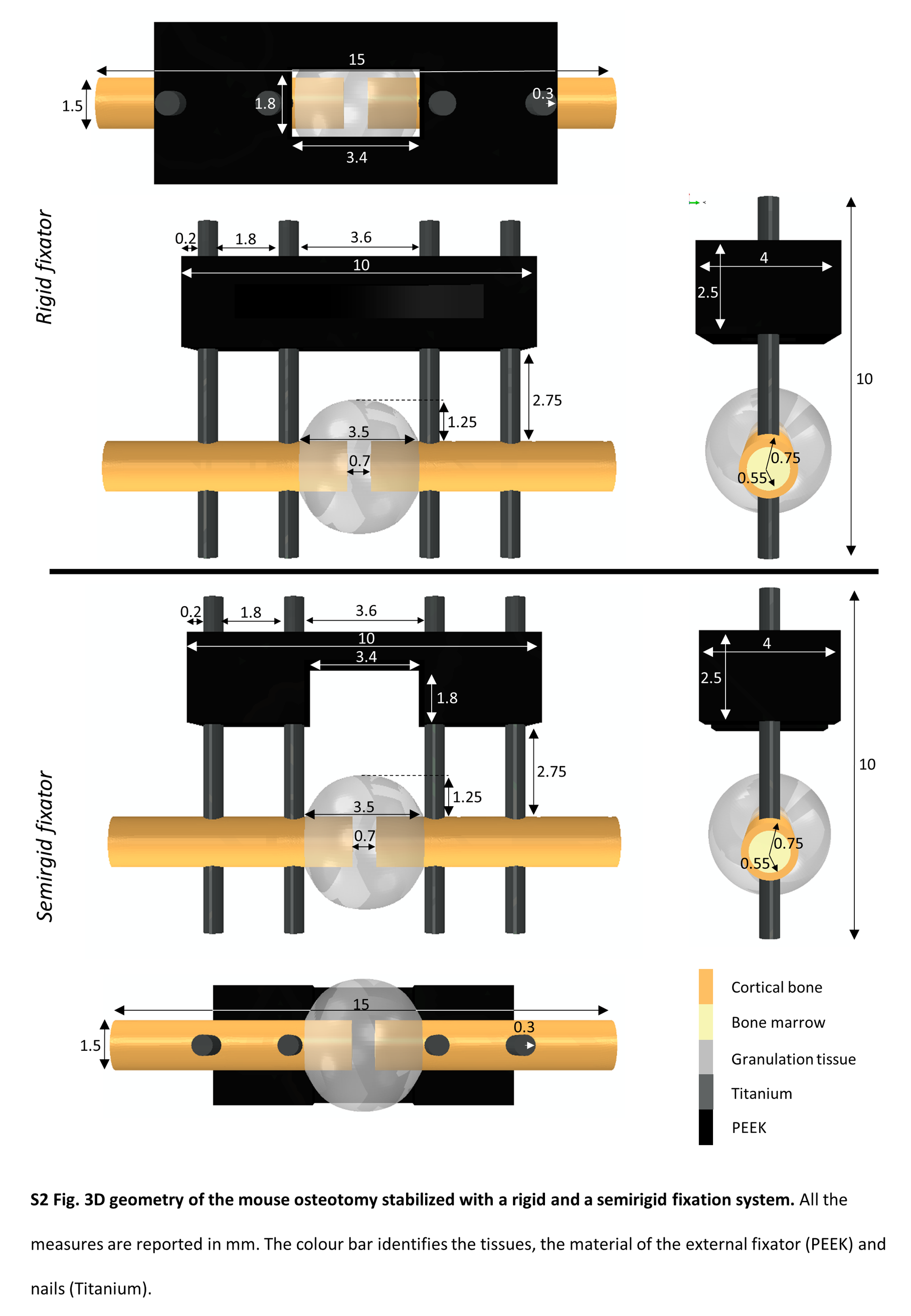

Supplement: S2 Fig — All the measures are reported in mm. The colour bar identifies the tissues, the material of the external fixator (PEEK) and nails (Titanium). (TIF) [file pcbi.1011647.s002.tif]

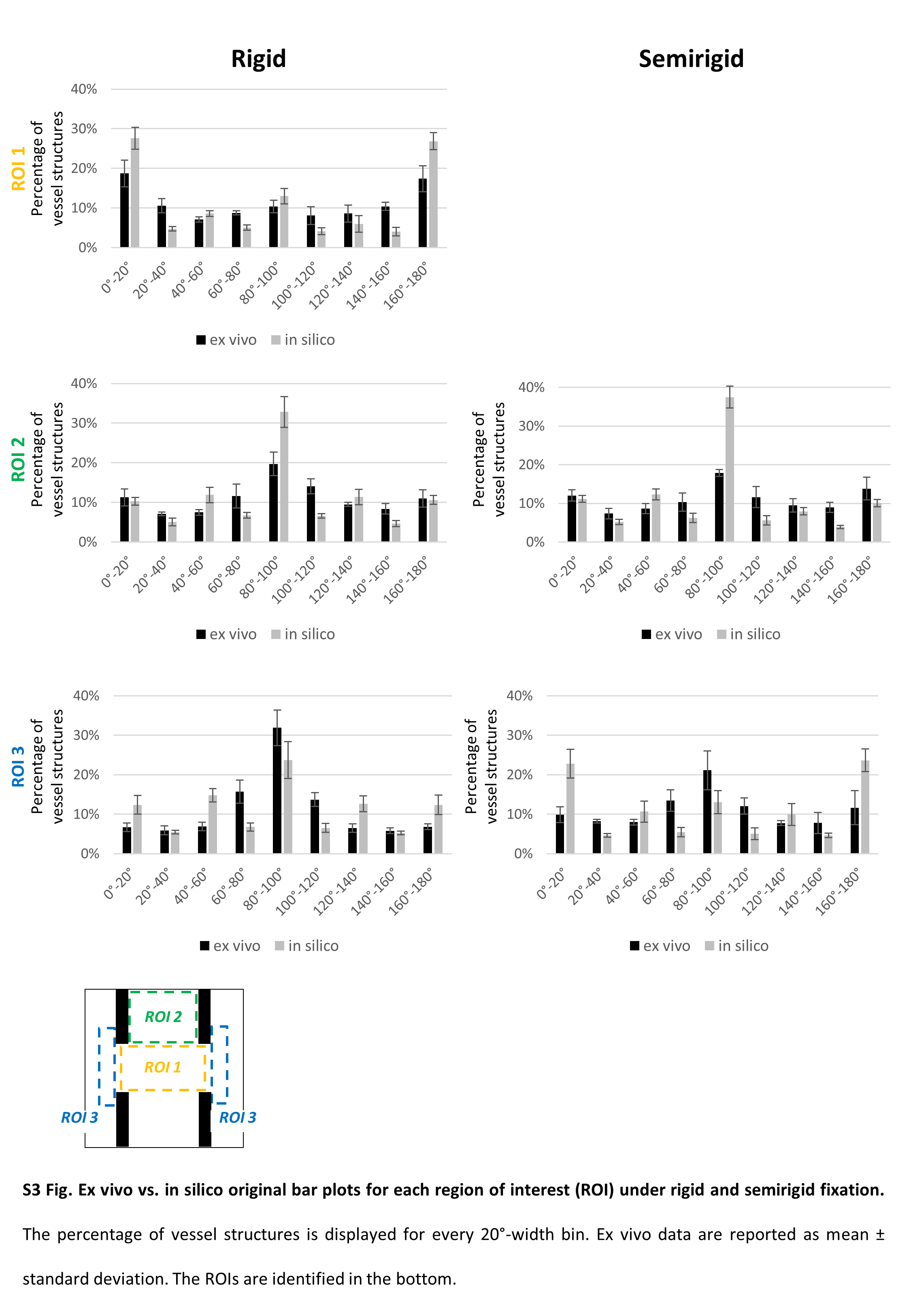

Supplement: S3 Fig — The percentage of vessel structures is displayed for every 20°-width bin. Ex vivo data are reported as mean ± standard deviation. The ROIs are identified in the bottom. (TIF) [file pcbi.1011647.s003.tif]

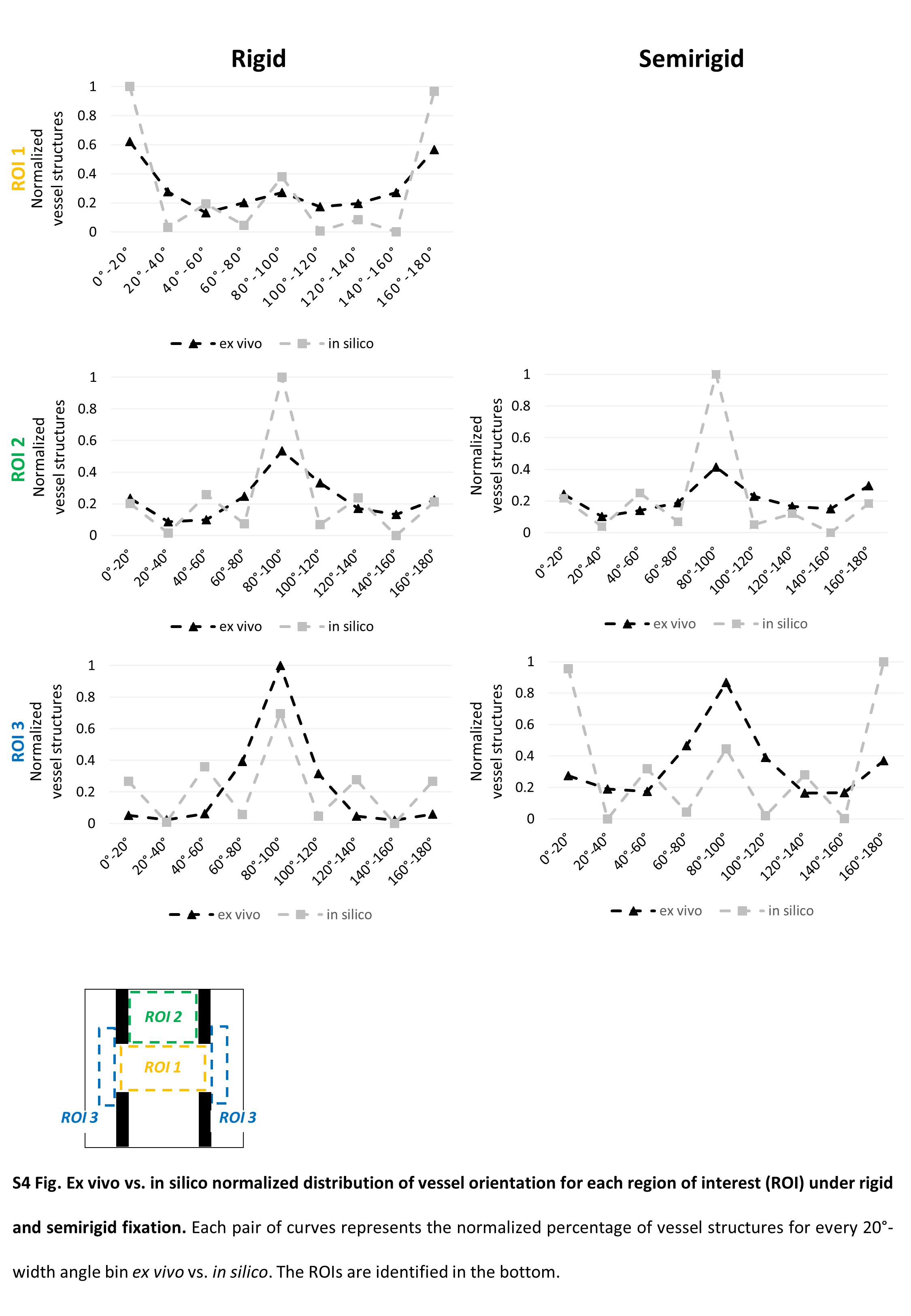

Supplement: S4 Fig — Each pair of curves represents the normalized percentage of vessel structures for every 20°-width angle bin ex vivo vs. in silico. The ROIs are identified in the bottom. (TIF) [file pcbi.1011647.s004.tif]

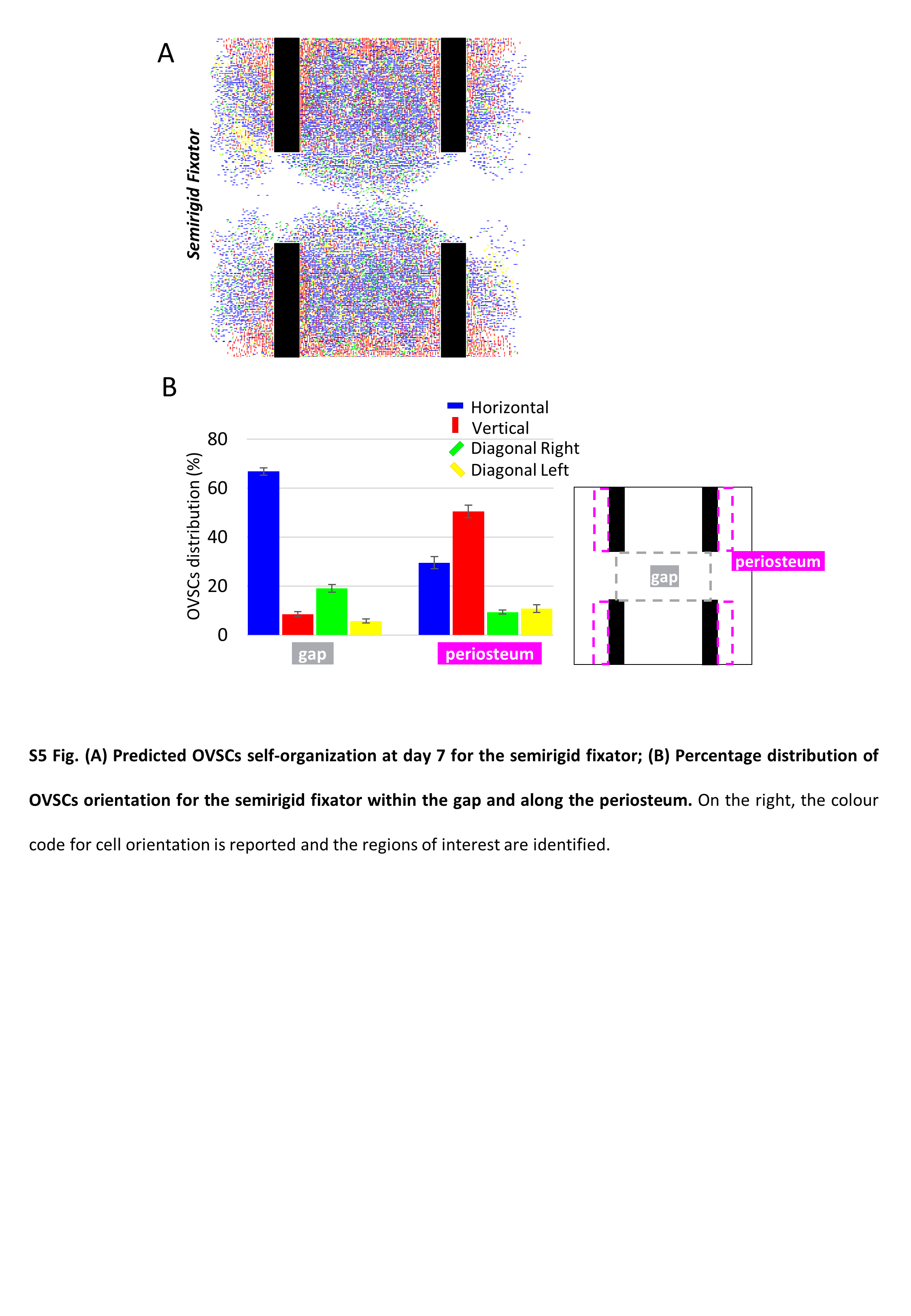

Supplement: S5 Fig — (A) Predicted OVSCs self-organization at day 7 for the semirigid fixator; (B) Percentage distribution of OVSCs orientation for the semirigid fixator within the gap and along the periosteum. On the right, the colour code for cell orientation is reported and the regions of interest are identified. (TIF) [file pcbi.1011647.s005.tif]

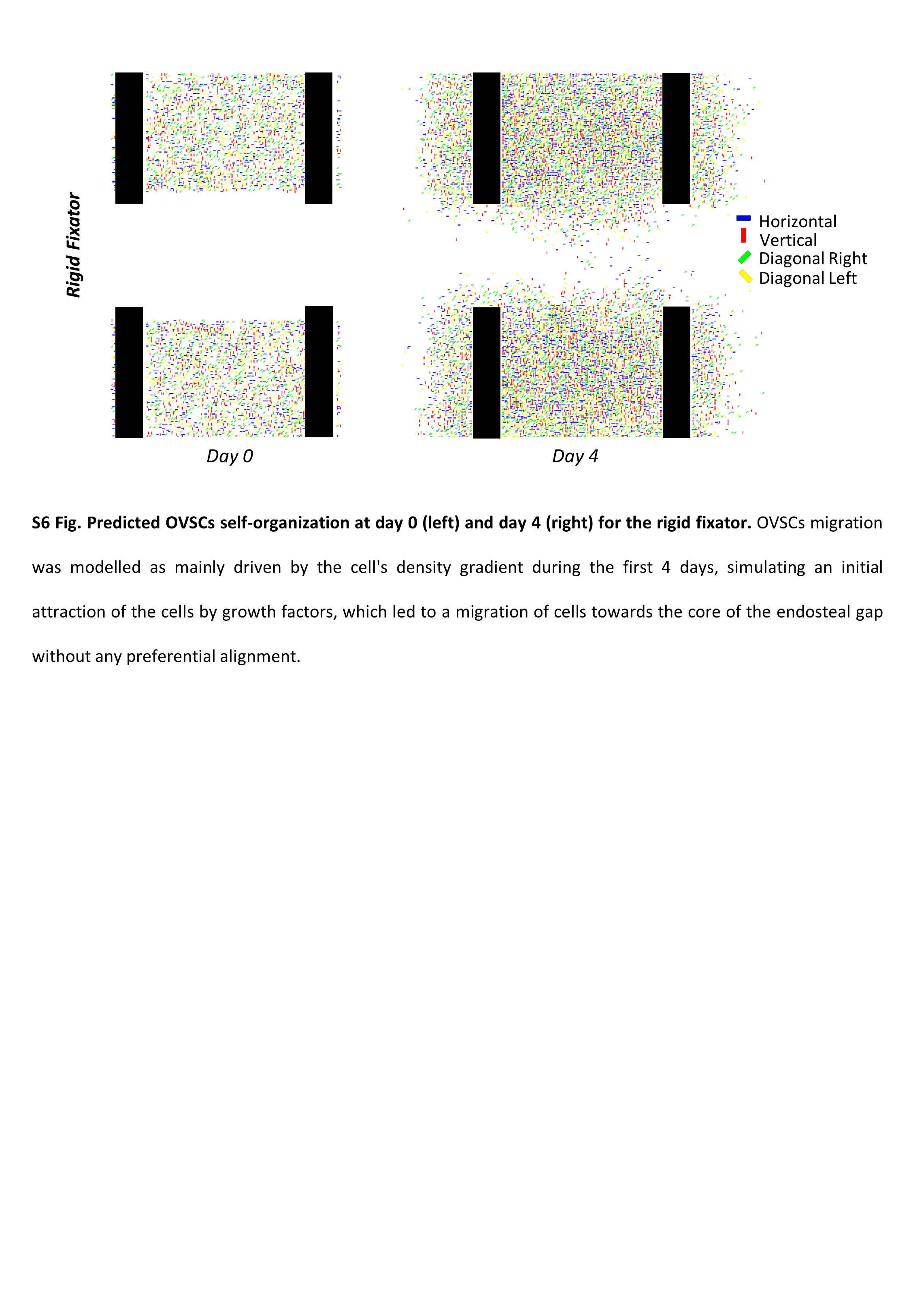

Supplement: S6 Fig — OVSCs migration was modelled as mainly driven by the cell’s density gradient during the first 4 days, simulating an initial attraction of the cells by growth factors, which led to a migration of cells towards the core of the endosteal gap without any preferential alignment. (TIF) [file pcbi.1011647.s006.tif]

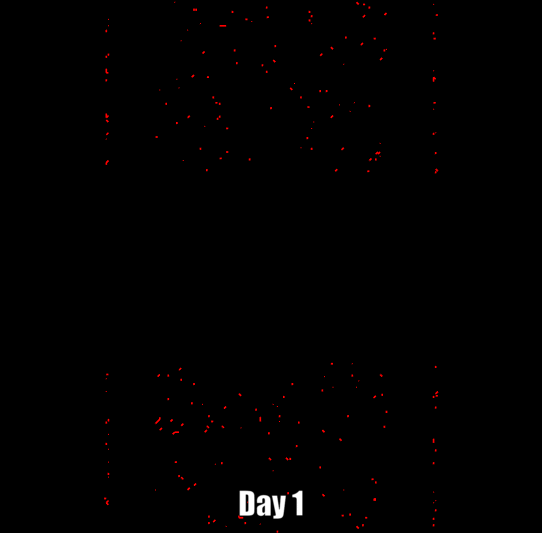

Supplement: S1 Movie — (GIF) [file pcbi.1011647.s009.gif]

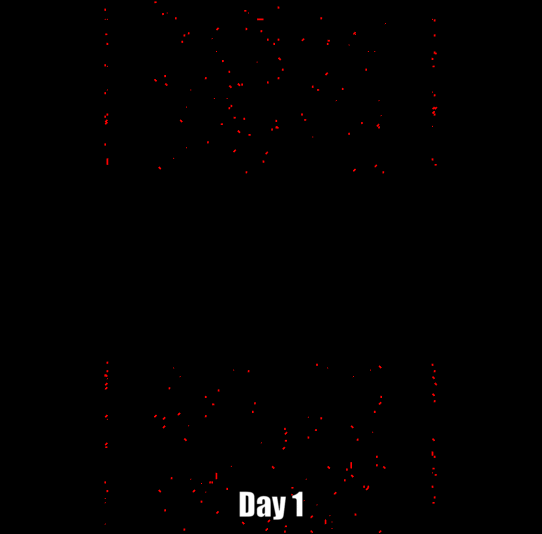

Supplement: S2 Movie — (GIF) [file pcbi.1011647.s010.gif]
