# Supplementary material for: External mechanical loading overrules cell-cell mechanical communication in sprouting angiogenesis during early bone regeneration
Source: PLoS Comput Biol. 2023 Nov 13;19(11):e1011647. doi: 10.1371/journal.pcbi.1011647 (PMC10681321; doi:10.1371/journal.pcbi.1011647)
Supplement: S1 File — (PDF) [file pcbi.1011647.s007.pdf]

S1 File

Displacement field due to sprout traction

We attempted to mimic the forces applied by the angiogenic sprout onto the extracellular matrix. Traction-force microscopy experiments (1) suggested that the sprout tip exerts radially distributed traction forces, while the whole sprout behaves like a dipole. However, previous studies have captured traction forces exerted by short sprouts, typically one tip cell only sprouting off from a cluster of ECs. This method did not allow to obtain a displacement field independent from the traction exerted by the spheroid of cells at the sprout base. To assign realistic traction forces in our models, data from a 3D traction force microscopy experiment on a longer sprout (2-3 cells) were provided by our collaborators (Mounir Benamar, Knaus Group). A simplified 2D FE model of a growing sprout onto a gelatin substrate was developed and two different configurations of traction force distribution were tested. We assumed that the leading tip cell of the sprout 1) behaves like an active force dipole and applies traction forces along the direction of vessel growth, thus changing direction at each iteration; 2) pulls on the substrate radially (Fig. S1a). Stalk cells were hypothesized to not contribute, in agreement with previous studies (2,3). Finite element simulations allowed computing the displacement field generated by the sprout that was then qualitatively compared with the experimental results (Fig. S1b). The displacement field generated by the sprout when pulling like an active force dipole resembled more the polarization of displacement vectors observed experimentally and the dipole configuration was therefore adopted for the current study.

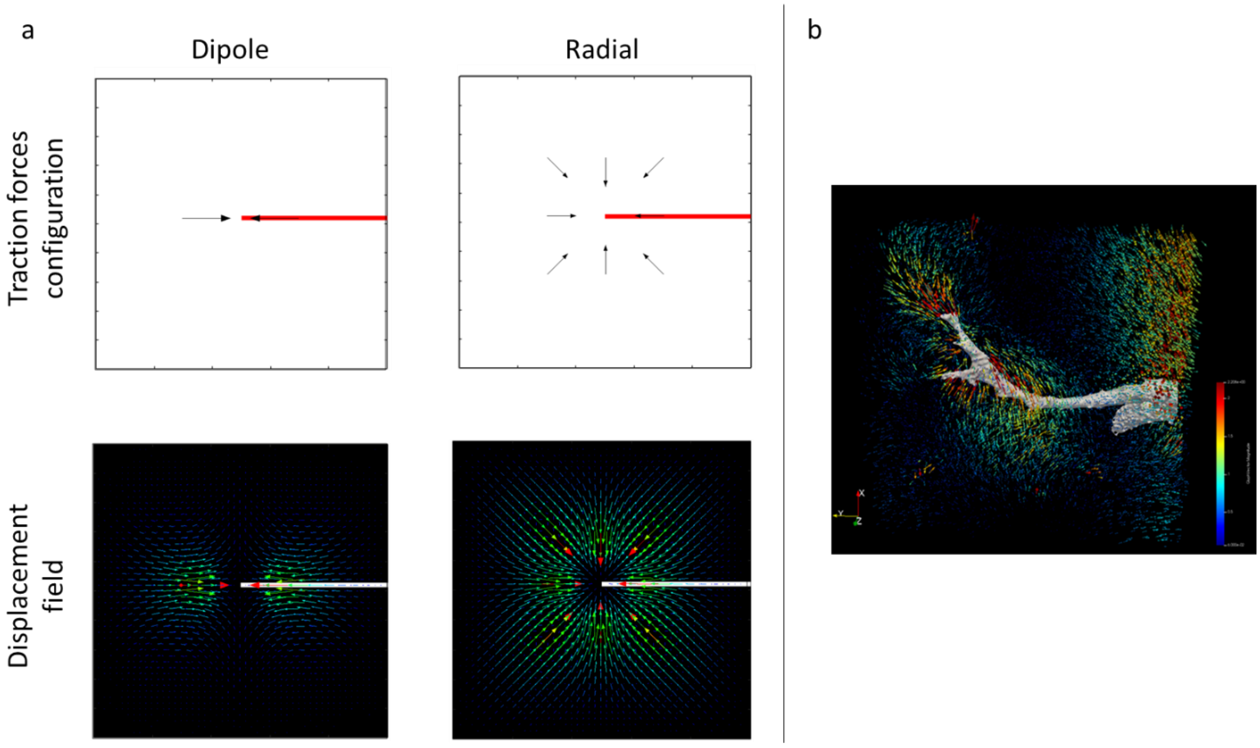

Fig. S1 a) Representation of traction forces assigned to the sprout tip in two different configurations and the associated predicted displacement fields below; b) Displacement field generated by the sprout from 3D traction force microscopy experiments.

References

1. Vaeyens MM, Jorge-Penas A, Barrasa-Fano J, Steuwe C, Heck T, Carmeliet P, et al. Matrix deformations around angiogenic sprouts correlate to sprout dynamics and suggest pulling activity. Angiogenesis. 2020;23(3):315-24.

2. Du Y, Herath SC, Wang QG, Wang DA, Asada HH, Chen PC. Three-Dimensional Characterization of Mechanical Interactions between Endothelial Cells and Extracellular Matrix during Angiogenic Sprouting. Sci Rep. 2016;6:21362.

3. Yoon C, Choi C, Stapleton S, Mirabella T, Howes C, Dong L, et al. Myosin IIA-mediated forces regulate multicellular integrity during vascular sprouting. Mol Biol Cell. 2019;30(16):1974-84.
