# Supplementary material for: External mechanical loading overrules cell-cell mechanical communication in sprouting angiogenesis during early bone regeneration
Source: PLoS Comput Biol. 2023 Nov 13;19(11):e1011647. doi: 10.1371/journal.pcbi.1011647 (PMC10681321; doi:10.1371/journal.pcbi.1011647)
Supplement: S2 File — (PDF) [file pcbi.1011647.s008.pdf]

S2 File

Parameter sweep analysis

A parameter sweep analysis was carried out in order to choose the combination of tip ECs probabilities to migrate following a specific rule (P1=persistency, P2=randomness, P3=mechanics) that allowed to better approximate experimental results in terms of vessel orientation distribution within the three regions of interest (gap or ROI1, bone marrow or ROI2, periosteal site or ROI3). Some constraints were imposed to identify the relevant parameter combinations (reported in Table S2):

- P1+P2+P3 = 1;
- P1, P2, P3 >= 0.2 in order to have a sufficient contribution of each parameter;
- P2 < 0.6 because with higher values, vessels resulted unrealistically short as compared to the histologies.

The analysis was performed for both the rigid and the semirigid fixation scenarios.

Table S2: all the parameter combinations considered and the associated model names.

Model name

	**1**	**2**	**3**	**4**	**5**	**6**	**7**	**8**	**9**	**10**	**11**	**12**	**13**	**14**
**P1**	0.3	0.4	0.5	0.6	0.2	0.3	0.4	0.5	0.2	0.3	0.4	0.2	0.3	0.2
**P2**	0.5	0.4	0.3	0.2	0.5	0.4	0.3	0.2	0.4	0.3	0.2	0.3	0.2	0.2
**P3**	0.2	0.2	0.2	0.2	0.3	0.3	0.3	0.3	0.4	0.4	0.4	0.5	0.5	0.6

All the combinations of parameters led to the experimentally observed preferential alignment of vessels within the different ROIs with an exception for ROI 3 (periosteal site) under semirigid fixator that was therefore excluded from the analysis (Figure S2 a). To select the best combination of probability values, the Mean Squared Error (MSE) between the computational and experimental distribution of vessel orientations within each ROI was computed. For each parameter combination, the MSE in every ROI and under both fixators were summed. The Total MSE for each parameter combination is displayed in Figure S2 b. The parameter combination that led to the minimum Total MSE across all ROI and fixation cases (rigid vs. semirigid) resulted in P1=0.4, P2=0.4, P3=0.2, thus Model 2 was selected as the best match.

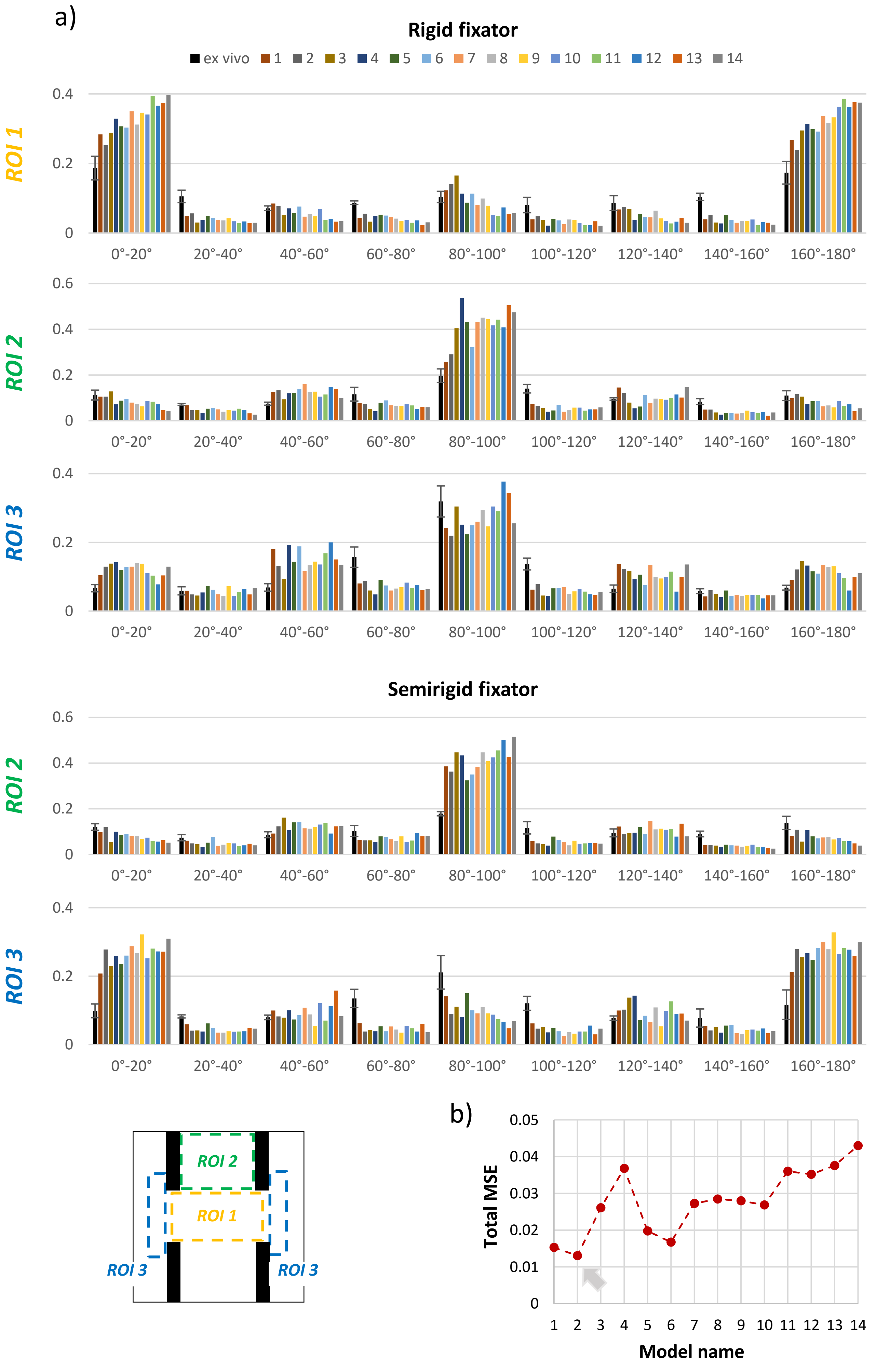
a)
Rigid fixator
ex vivo 1 2 3 4 5 6 7 8 9 10 11 12 13 14
ROI 1
ROI 2
ROI 3
0 0.2 0.4 0.6
0°-20° 20°-40° 40°-60° 60°-80° 80°-100° 100°-120° 120°-140° 140°-160° 160°-180°
Semirigid fixator
ROI 2
ROI 3
ROI 1
b)
Total MSE
0 0.01 0.02 0.03 0.04 0.05
1 2 3 4 5 6 7 8 9 10 11 12 13 14
Model name

Fig. S2 a) Vessel orientation analysis for all the combinations of probability values (P1, P2, P3) across the three regions of interest for both fixators; b) Total MSE for each parameter combination. The minimum is indicated by the gray arrow.
