# Supplementary material for: External mechanical loading overrules cell-cell mechanical communication in sprouting angiogenesis during early bone regeneration
Source: PLoS Comput Biol. 2023 Nov 13;19(11):e1011647. doi: 10.1371/journal.pcbi.1011647 (PMC10681321; doi:10.1371/journal.pcbi.1011647)
Supplement: Attachment — Submitted filename: R_Manuscript_PCOMPBIOL-D-23-00666.pdf [file pcbi.1011647.s011.pdf]

The manuscript entitled "Extrinsic mechanical loading overrules cell-cell mechanical communication in sprouting angiogenesis during early bone regeneration" presents an experimentally motivated computational model of tissue vascularisation during early bone healing. The study focuses on investigating the interplay between external mechanical loads, which depend on physical activity and the fracture fixation system, and internal strains generated by cell activity during bone regeneration. At the cellular scale, the model employs an agent-based framework to simulate the growth of vascular sprouts using the snail-trail approach, as well as stromal cell migration and orientation. At the tissue scale, the authors utilise the Finite Element Method to analyse the strains induced within the surrounding tissue, resulting from both external physiological loading and traction forces generated by cells (including endothelial and stromal cells). This multiscale computational framework demonstrates how extrinsic mechanical loading influences angiogenesis, outweighing the impact of cell-cell mechanical communication during early bone regeneration.

The problem addressed in this work is of considerable interest for improving our understanding of cell behaviour during bone healing and potential clinical applications for designing improved fracture fixators. The model is based on experimental observations, and simulation results are compared to the available *ex vivo* experimental data. The authors also made available the code of their computational model, which is a valuable resource. Despite the manuscript's merits, I have identified several major issues that should be addressed before publication. These points are described in more detail below, and I believe that resolving them will improve the quality of the manuscript. Once these concerns are adequately addressed, I am pleased to recommend the manuscript for publication.

Major issues:

1. While the modelling results offer new insights into the field, I suggest that the authors revise the language of the Introduction and Discussion sections to improve the readability of the manuscript.

2. I believe that the manuscript will benefit from a clearer conclusion based on the obtained simulation results regarding what loading (or combination of loadings) is predicted to promote vascularisation and bone regeneration. From my understanding after reading the manuscript, a semiflexible fixation system leads to delayed vascular growth. However, it is unclear whether the rigid fixator or unloaded scenario favours vascular growth. Likewise, under rigid/semirigid fixation, the vascular sprouts are more organised in their alignment, but this does not necessarily imply more efficient vascularisation. In fact, the unloaded scenario, in which stromal cell traction forces are knocked out (Fig. 8B), visually appears to exhibit more favorable vessel distribution (as compared to Figs. 8A and 6A).

3. I would like to suggest a modification in the representation of simulation results regarding vessel alignment, as shown in Fig. 4B. The current representation may lead to confusion or misinterpretation. While I understand that the figure includes a breakdown of vessel orientation into three alignment directions (horizontal, vertical, and other), the percentages presented for each category might not accurately reflect the preferential orientation as described in the text. For instance, in the case of the rigid fixator (ROI1, *ex vivo*), roughly saying that horizontal alignment is 20% and other alignment is 10% might give the impression that vertical alignment constitutes the preferential orientation at 70%. This interpretation contradicts the explanation provided in the text, which suggests a preferential horizontal alignment. Moreover, it is difficult to connect Fig. 4B to the original distribution of vessel orientations from Fig. S4. To improve clarity and avoid ambiguity, I recommend that the authors include all three alignment categories in the histograms.

   In addition to the concern raised regarding the representation of the comparison between vessel orientation from *ex vivo* samples and simulations, it is challenging to accept that they are in agreement based on the current presentation. This raises another question of whether the results from the computational modelling are from single realisations or averaged over multiple runs. Likewise, have the authors tried to perform parameter sweeps or check different functional forms for the mechano-response for sprout tips to improve the agreement with the experimental data? Can the authors clarify this within the text or add this analysis to their study if it has not been performed yet?

   I suggest averaging all simulation results over multiple realizations and reporting them with the corresponding error bars (if the error is already small for a few replicas due to a large number of vessels, then this suggestion is irrelevant).

Minor issues:

4. Could the authors clarify what they mean by *dedicated* experiments?

5. I would like to suggest adding a schematic illustrating the differences between rigid and semirigid fracture fixators (perhaps as a supplementary figure).

6. On line 41, “should be used” is perhaps too strong; the authors might want to soften this.

7. Can the authors indicate the meaning of the abbreviation ROI when it appears for the first time on line 288?

8. On lines 122 and 455 should it be *ex vivo* instead of *in vivo*?

9. On line 165, the authors mention two distinct regions of interest but then all the simulation results are presented for three regions.

10. In Fig 1, the tip cell is shown with filopodia on two opposite sides of the cell, while it is known that tip cells extend filopodia only at the front of the cell in the direction of migration. I would like to recommend the authors to correct this. In addition, in the same Fig. 1, can the authors explicitly annotate (or explain in the caption) what green arrows indicate (Ux, Uy?)?

11. In Table 1, could the authors indicate what P_1, P_2, and P_3 represent? I understand that those are different migrational probabilities, but adding this information explicitly will improve readability. Likewise, do lines 187-188 belong to the Table 1 caption? (In case they do, the remark is irrelevant.)

12. In Fig. 2, I would like to recommend the authors indicate migration probabilities P_1 and P_2 instead of $R < 4$ and $4 \leq R \leq 6$, as those are parameters of the model and potentially can vary (and they indeed do later in the knockout mechano-response for tip cells).

13. Around lines 257-261, where the rules for the stromal cell migration and orientation are described, could the authors clarify why they assume random migration direction for these cells if they are known to follow up the gradient of stiffness?

14. In the numerical scenario of inhibited mechano-response of tip cells, when $P_3 = 0$, what are the corresponding values of P_1 and P_2?

15. On lines 301-302, “the shape of a line differs from a straight line” perhaps refers to the shape of a vessel segment. Could the authors revise this?

16. Line 320 states “immediately after surgery” but the figures show vessel distribution on the 7th day after the intervention.

17. I would like to suggest making the colour bar in Fig. 3A longer and indicating the key strain values for the assumptions of the model (5%, 10%, and 30%).

18. It might be helpful to add the strain distribution for the unloaded scenario with traction forces coming from cell activity only (to compare it to Fig. 3A).

19. Could the authors clarify within the text how the orientation of stromal cells is related to the alignment of collagen fibers (e.g. around lines 370-372 and 477-479)? It is explained in more detail close to the end of Discussion, but for interpretation of the results, as they are reported, it would be helpful to have these details earlier.
