# Supplementary material for: External mechanical loading overrules cell-cell mechanical communication in sprouting angiogenesis during early bone regeneration
Source: PLoS Comput Biol. 2023 Nov 13;19(11):e1011647. doi: 10.1371/journal.pcbi.1011647 (PMC10681321; doi:10.1371/journal.pcbi.1011647)
Supplement: Attachment — Submitted filename: Response to Reviewers.pdf [file pcbi.1011647.s012.pdf]

We would like to express our gratitude to the reviewers for their valuable feedback, which have undoubtedly strengthened the quality of our manuscript. In what follows, we provide a detailed point-by-point reply to each question raised.

**Reviewer #1:** Dazzi et al. develop an in silico model to predict mechano-regulation of sprouting angiogenesis in early bone healing. Computational growth predictions were compared to in vivo findings of a mouse osteotomy model, which was either stabilized with a rigid or a semi-rigid fixation system. Computational modelling demonstrates that mechanical strains and strain directions can explain vessel patterning at the onset of bone healing.
Furthermore, with rigid fixation loss of endothelial tip cell mechano-responsiveness resulted an altered vessel organization, which is agreement with experimental findings on angiogenic processes in other vascular beds.
In particular, the authors show that only in the absence of rigid bone fixation, traction forces generated by the surrounding OVSCs might regulate endothelial sprouting behavior.
Although the computational model has several limitations (which are well discussed) the study shows some interesting findings.

Answer: we would like to thank the reviewer for the careful reading of the manuscript.

Specific points:
• From a biomechanical perspective the manuscript might in some parts confuse the reader. With a focus on endothelial mechano-regulation on a cellular level, intrinsic loads/forces are generated from within the endothelial cell, while extrinsic loads/forces (on the endothelial cell) are exerted by the surrounded cells (here termed OVSCs). Throughout the manuscript, the authors should try to clarify extrinsic loads on a cellular versus tissue level, e.g. external instead of extrinsic.

Answer: we thank the reviewer for the suggestion. We agree the terms "intrinsic" and "extrinsic" might be misleading and we have changed them to "cell-induced" and "external/externally applied" throughout the manuscript, including the title.

• Linear elastic properties from previous studies have been utilized to feed the computational model. How have those values been experimentally measured? Other studies (e.g. Ghiasi MS, Chen JE, Rodriguez EK, Vaziri A, Nazarian A. Computational modeling of human bone fracture healing affected by different conditions of initial healing stage. BMC Musculoskelet Disord. 2019 Nov 25;20(1):562. doi: 10.1186/s12891-019-2854-z. PMID: 31767007; PMCID: PMC6878676) demonstrate quite variable elastic modulus values for granulation tissue. How would stiffness values for the tissue affect the here developed model?

Answer: we thank the reviewer for the questions and for bringing this point to our attention. The elastic modulus of cortical bone was measured experimentally in rat femurs by Jørgensen et al. (1). The elastic modulus of the bone marrow has been determined by Hosokawa and Otani (2, 3), by means of ultrasound. Granulation tissue is the least characterized tissue. We assigned to the granulation tissue an elastic modulus of 0.2 MPa, originally estimated by Lacroix and Prendergast (4) to replicate the experimental set-up of Claes et al. (5) and Goodship and Kenwright (6). This choice was motivated by the need to be consistent with the mouse osteotomy model developed by Borgiani et al. (7) since displacement boundary conditions for the current study were extracted from that model. Moreover, an elastic modulus of 0.2 MPa was assigned to granulation tissue by many previous computational studies investigating bone regeneration (8-13). These models could predict the tissue formation at several time points when compared to *in vivo* experiments, also in mice (7, 11). We would like to mention that the elastic modulus of granulation tissue was measured experimentally, to our knowledge, only by Leong and Morgan (14). By performing nano indentation on rats femurs 35 days post-fracture, they found an elastic modulus of around 1 MPa. However, here we are modelling the first week post-fracture and the elastic modulus of the granulation tissue is likely to be lower at the onset of healing. We have added all the experimental references to Table I.
In addition, we have added a paragraph in the Discussion section (lines 590-594):

“In order to apply displacement boundary conditions consistent with the original 3D model developed by Borgiani et al. (7), we assigned identical material properties to the tissues as those reported in that study. However, the granulation tissue mechanical properties remain poorly investigated, especially at the onset of bone healing. Once experimental data are available, a more realistic description of its mechanical behaviour should be included in the model.”

• The authors describe and discuss that under unloading conditions and in the absence of OVSC traction forces sprout patterning was impacted and led to straighter vessels without any preferential orientation. However, the (patho)biological consequences of this finding are not discussed.

Answer: Thank you for the comment. We included the aforementioned analysis (knock out of OVSCs traction forces under unloading conditions) as a mean of comparison for the unloading scenario. In particular, we wanted to show that OVSCs affect sprout patterning once the prevailing external load is removed by inducing a certain vessel alignment within the gap along the bone axis direction. This preferential alignment disappears, and vessels returned randomly organized once the OVSCs forces are removed. These results show that OVSCs can organize the vascular network through OVSCs traction forces-induced strains within the ECM.
To clarify why we included this analysis, we have added a sentence to the Materials and Methods section (lines 306-308):

“As a control, OVSCs traction forces were inhibited under unloading conditions and simulation results in the presence and absence of OVSCs traction forces were compared.”

In addition, following the reviewer’s suggestion, we have added more details on the biological implications of this finding in the Discussion section (lines 581-583):

“Taken together, these findings under unloading conditions propose that the strains induced by OVSCs traction forces within the ECM act as a regulator of vessel organization in the absence of high external loads, corroborating previous experimental observations (15, 16).”

• Further, the authors claim that upon OVSC traction force inhibition, the only strains sensed by tip ECs are those generated by themselves along the sprout growth direction (Line 516/517). What about the passive matrix/tissue stiffness provided by the presence of the OVSCs? The reviewer believes this statement is simplified from a modelling perspective but does not agree with the biological system.

Answer: we thank the reviewer for the comment. We agree with the reviewer that OVSCs contribute to the passive matrix stiffness even in the absence of cell contractility. However, we did not take this element into account in the current study. As mentioned by the reviewer, the reported statement refers to the strains sensed by the tip ECs in the developed computer model.
We clarified this by adjusting the sentence (line 578) as follows:

“the only strains sensed by tip ECs in the developed model are those generated by themselves along the sprout growth direction”

• External fixation of bone fractures is still the most common treatment strategy. Can the authors explain how mechanical tuning beyond standard fixation could look like?

Answer: we thank the reviewer for the input. We assume the reviewer refers to the last part of the Discussion (lines 628-636). We did not intend to suggest overcoming standard fixation, but to invite the research community to focus on mechanical signals to induce a proper angiogenic response during bone regeneration. Several cell- and chemical-based treatments have been developed and applied to modulate angiogenesis during bone regeneration (17) but these are often associated with unwanted side effects. Here, we show that angiogenesis responds to mechanical signals during early bone regeneration and that sprout patterning can be explained by a combination of principal strain levels and orientation. This knowledge can be used to mechanically optimize treatment strategies, even existing standard ones, towards an appropriate angiogenic response. For example, the stiffness of an

external fixator could be adjusted so as to induce principal strain levels and orientation within the healing region that are favourable for sprouting.
We have modified the text as follows (lines: 628-636):

"The knowledge gained in this study could advance the development of regeneration strategies based on the mechanical control of angiogenesis. Devices like stabilization systems and scaffolds should be mechanically optimized to modulate ECs mechano-response and thus promote microvascular invasion at the onset of healing. For example, the stiffness of standard external fixation systems could be adjusted throughout the treatment phase so as to induce principal strain levels and orientations within the healing region that are favourable for sprouting. Tuning sprouting through mechanics at the tissue and cell level would enable a more targeted and controlled therapy approach for the regeneration of bone, without the unwanted side effects that can occur with cell- and growth factor- based treatments."

• There is little to no information on how many independent mouse experiments were performed, quantifications of in vivo/ex vivo data are missing.

Answer: thank you for the comment. We added more details to the Materials and Methods section (lines 130-131):

"Eight female mice aged 9-10 weeks underwent a diaphyseal osteotomy on the left femur stabilized by an external rigid (n=4) or semirigid (n=4) fixation system (MouseExFix 100% and 50%, RISystem, S1 Fig.)."

All ex vivo histologies were analysed in terms of vessel density and vessel orientation distribution within three regions of interests distinguishing between the gap, the bone marrow, and the periosteum. More details can be found in the paragraph ("Quantification of microvascular network parameters", lines: 310-328). Quantification results can be found in the Results section, Figures 3 and 4.

• Most of the supplementary figures are lacking figure legends. Those should be added.

Answer: we thank the reviewer for the comment and have included all the figure legends in the supplementary figures.

• Figure 3D: "By Julia Mehl" should be removed.

Answer: thanks for the comment, we have removed it.

• Figure 5C: The figure contains previously published data. The Creative Commons public licenses should be provided.

Answer: we thank the reviewer for the comment. The figure was generated from an experiment performed in our Institute by Ansgar Petersen, who provided us with the original image. We have now included a modified version of the image (Figure 5A). In addition, a description is provided in the text and figure caption.

• Line 482: To isolate "various intrinsic and extrinsic mechanical signals" seems exaggerated and not precise.

Answer: thank you for the comment. We have changed the sentence as follows (line 542):
"The goal was to isolate mechanical signals acting at the cell and tissue level to better understand their role in early sprout patterning, which is not feasible experimentally."

**Reviewer #2**: The manuscript entitled "Extrinsic mechanical loading overrules cell-cell mechanical communication in sprouting angiogenesis during early bone regeneration" presents an experimentally motivated computational model of tissue vascularisation during early bone healing. The study focuses on investigating the interplay between external mechanical loads, which depend on physical activity

and the fracture fixation system, and internal strains generated by cell activity during bone regeneration. At the cellular scale, the model employs an agent-based framework to simulate the growth of vascular sprouts using the snail-trail approach, as well as stromal cell migration and orientation. At the tissue scale, the authors utilise the Finite Element Method to analyse the strains induced within the surrounding tissue, resulting from both external physiological loading and traction forces generated by cells (including endothelial and stromal cells). This multiscale computational framework demonstrates how extrinsic mechanical loading influences angiogenesis, outweighing the impact of cell-cell mechanical communication during early bone regeneration.
The problem addressed in this work is of considerable interest for improving our understanding of cell behaviour during bone healing and potential clinical applications for designing improved fracture fixators.
The model is based on experimental observations, and simulation results are compared to the available ex vivo experimental data. The authors also made available the code of their computational model, which is a valuable resource. Despite the manuscript's merits, I have identified several major issues that should be addressed before publication. These points are described in more detail below, and I believe that resolving them will improve the quality of the manuscript. Once these concerns are adequately addressed, I am pleased to recommend the manuscript for publication.

We thank the reviewer for the careful reading of the manuscript and appreciating the relevance of the research topic.

Major issues:

1. While the modelling results offer new insights into the field, I suggest that the authors revise the language of the Introduction and Discussion sections to improve the readability of the manuscript.

Answer: we thank the reviewer for the suggestion and we have revised the language adopted in the Introduction and Discussion sections.

2. I believe that the manuscript will benefit from a clearer conclusion based on the obtained simulation results regarding what loading (or combination of loadings) is predicted to promote vascularisation and bone regeneration. From my understanding after reading the manuscript, a semiflexible fixation system leads to delayed vascular growth. However, it is unclear whether the rigid fixator or unloaded scenario favours vascular growth. Likewise, under rigid/semirigid fixation, the vascular sprouts are more organised in their alignment, but this does not necessarily imply more efficient vascularisation. In fact, the unloaded scenario, in which stromal cell traction forces are knocked out (Fig. 8B), visually appears to exhibit more favourable vessel distribution (as compared to Figs. 8A and 6A).

Answer: we thank the reviewer for the comment. This study was motivated by *in vivo* experiments where we have seen a significant preferential alignment of vessels in different regions of interests under both a rigid and a semirigid fixator and a significantly reduced vascularization under semirigid fixation. From the experimental results, we can already deduce that a rigid fixation is superior to the semirigid one. Our goal was to develop a literature informed computer model that could help explain what mechanical signals lead to the specific sprout patterning observed in both cases.
Moreover, while external fixators with a different stiffness are employed clinically, clinical studies have shown in recent years that unloading leads to delayed healing (18, 19). Therefore, the purpose of the unloading scenario was not to replicate a clinically relevant case or suggest a better approach to treat fractures, but to isolate the cell-induced mechanical forces from the high external loads to investigate the cell-cell mechanical communication. This is why simulations results under unloading conditions are discussed against the available *in vitro* literature on the topic.

We tried to clarify this in the Discussion section (lines 564-568):

"In recent years, clinical studies have shown that unloading hinders bone healing (18, 19). Therefore, the purpose of the unloading scenario was not to replicate a clinically relevant case or suggest a better approach to treat fractures, but to isolate the cell-induced mechanical forces from the high external loads to investigate the cell-cell mechanical communication. After the cell-induced forces were

isolated, hints of the ECs-OVSCs mechanical interaction emerged and results are discussed in the context of the available *in vitro* literature."

3. I would like to suggest a modification in the representation of simulation results regarding vessel alignment, as shown in Fig. 4B. The current representation may lead to confusion or misinterpretation. While I understand that the figure includes a breakdown of vessel orientation into three alignment directions (horizontal, vertical, and other), the percentages presented for each category might not accurately reflect the preferential orientation as described in the text. For instance, in the case of the rigid fixator (ROI1, ex vivo), roughly saying that horizontal alignment is 20% and other alignment is 10% might give the impression that vertical alignment constitutes the preferential orientation at 70%. This interpretation contradicts the explanation provided in the text, which suggests a preferential horizontal alignment. Moreover, it is difficult to connect Fig. 4B to the original distribution of vessel orientations from Fig. S4. To improve clarity and avoid ambiguity, I recommend that the authors include all three alignment categories in the histograms.

Answer: thank you for the suggestion. We agree that the bar plot was difficult to interpret, and we have reported all three orientation categories in the updated manuscript.

In addition to the concern raised regarding the representation of the comparison between vessel orientation from ex vivo samples and simulations, it is challenging to accept that they are in agreement based on the current presentation. This raises another question of whether the results from the computational modelling are from single realisations or averaged over multiple runs. Likewise, have the authors tried to perform parameter sweeps or check different functional forms for the mechano-response for sprout tips to improve the agreement with the experimental data? Can the authors clarify this within the text or add this analysis to their study if it has not been performed yet?

Answer: we thank the reviewer for the suggestion. Initially, we have tested a few parameters combinations only to identify the values of P1,P2 and P3 (probabilities of tip ECs to migrate according to a specific rule). However, we think this suggestion can really improve the manuscript and the fidelity of the *in silico* model. Therefore, we have performed a complete parameter sweep analysis to find the combination of parameters (P1,P2,P3) that better replicate experimental results. More details can be found in the Supporting Information, File S4.

We have mentioned the parameter sweep analysis at the lines 224-227 of the Materials and Methods section:

"To estimate the values of these probabilities, they were changed systematically through a parameter sweep analysis. The combination of values that better matched experimental data in terms of sprout patterning was selected and is reported in Table I. More details on the parameter sweep analysis are provided in the Supporting Information (S4 File)."

I suggest averaging all simulation results over multiple realizations and reporting them with the corresponding error bars (if the error is already small for a few replicas due to a large number of vessels, then this suggestion is irrelevant).

Answer: we thank the reviewer for the suggestion. We have not shown the error bars for the in silico results in the previous version of the manuscript because the error over multiple runs was negligible for the baseline models under rigid and semirigid fixator. However, after performing the parameter sweep analysis, the optimal combination of probability values resulted in an increased probability for ECs to migrate in a random direction (P2). This leads to some variance in the distribution of vessel orientation over multiple realizations. Therefore, in this updated version of the manuscript, we present the in silico results as averaged over 6 realizations for each simulation. Standard deviation is reported as dispersion measure.

We have provided this information at the lines 310-313 of the Materials and Methods section:

“Due to the stochastic component included in the initial seeding and in the migration rules for both ECs and OVSCs, six realizations for each simulation were performed. The realization closest to the mean is reported as representative image for each simulated scenario. *In silico* and *ex vivo* results are reported as mean ± standard deviation. “

Minor issues:
4. Could the authors clarify what they mean by dedicated experiments?

Answer: we intend that experiments have been carried out with a specific research question in mind, that is to investigate the mechanical regulation of angiogenesis during early bone healing. To avoid confusion, we have rephrased the sentences by removing “dedicated”.

5. I would like to suggest adding a schematic illustrating the differences between rigid and semirigid fracture fixators (perhaps as a supplementary figure).

Answer: thank you for the suggestion. We have added a schematic of the rigid and semirigid fixators to the Supporting Information, S1 Fig.

6. On line 41, “should be used" is perhaps too strong; the authors might want to soften this.

Answer: thank you for the comment. We changed “should” to “could”.

7. Can the authors indicate the meaning of the abbreviation ROI when it appears for the first time on line 288?

Answer: thank you very much, we have fixed it.

8. On lines 122 and 455 should it be ex vivo instead of in vivo?

Answer: yes, thank you.

9. On line 165, the authors mention two distinct regions of interest but then all the simulation results are presented for three regions.

Answer: thank you for the comment. We apologize for the confusion. On that line, we referred to OVSCs organization. While we quantified sprout patterning in three regions of interest, the OVSCs organization was quantified in two regions of interest due to the availability of experimental data. We tried to clarify this by rephrasing that paragraph (lines 165-170):

“The predicted sprout patterning on day 7 was compared to the *in vivo* experimental results at the same time point. OVSCs organization on day 7 was analysed in terms of cell orientations and compared to available experimental observations of collagen organization during the early stages of healing. To our knowledge, there are no experimental studies that have quantified OVSCs organization during the early stages of bone healing. However, collagen fibres have been reported to relate well with stromal cell orientation (20, 21).”

In addition, to improve clarity, we moved the sentences referring to the two regions of interest to the Results section where the experimental image is actually reported, lines 401-404.

10. In Fig 1, the tip cell is shown with filopodia on two opposite sides of the cell, while it is known that tip cells extend filopodia only at the front of the cell in the direction of migration. I would like to recommend the authors to correct this. In addition, in the same Fig. 1, can the authors explicitly annotate (or explain in the caption) what green arrows indicate (Ux, Uy?)?

Answer: thank you for the correction on the filopodia representation, we have now changed it.
In addition, we have explained what green and purple arrows indicate in the Fig.1 caption.

11. In Table 1, could the authors indicate what P1, P2, and P3 represent? I understand that those are different migrational probabilities, but adding this information explicitly will improve readability. Likewise, do lines 187-188 belong to the Table 1 caption? (In case they do, the remark is irrelevant.)

Answer: thank you for the suggestion, we modified Table I.
Yes, those lines belong to the table 1 caption.

12. In Fig. 2, I would like to recommend the authors indicate migration probabilities P1 and P2 instead of R < 4 and 4 _ R _ 6, as those are parameters of the model and potentially can vary (and they indeed do later in the knockout mechano-response for tip cells).

Answer: thank you for the recommendation, we adjusted Figure 2 and its caption.

13. Around lines 257-261, where the rules for the stromal cell migration and orientation are described, could the authors clarify why they assume random migration direction for these cells if they are known to follow up the gradient of stiffness?

Answer: thank you for the question. We assumed a random migration with a preference for less densely populated locations during the first days post-surgery only, in order to ensure a directed migration of cells towards the osteotomy gap, as observed experimentally. A rule based on the local stiffness was added from day 4. We tried to clarify by rewriting the lines 268-275 as follows:

"During the first days post-surgery, the release of growth factors at the healing site, like Transforming Growth Factor Beta (22), is known to attract OVSCs. To simulate this directed migration of OVSCs towards the osteotomy gap, OVSCs migration was modelled as a random walk with a preference for less densely populated locations during the first 4 days post-surgery. In addition, OVSCs have been reported to probe the local deformation along the dipole direction (23) and to change their position and dipole orientation towards areas of highest stiffness (*durotaxis*) (24). Therefore, after 4 days, OVSCs migration was biased by a rule dependent on local stiffness, based on Checa et al. (2015) (25)."

14. In the numerical scenario of inhibited mechano-response of tip cells, when P3 = 0, what are the corresponding values of P1 and P2?

Answer: thanks for the question. The inhibition of tip ECs mechano-responders, such as ROCK, was shown to disrupt the alignment of vessel structures under cyclic stretch (26). Therefore, the tip ECs probability of migration in a random direction was increased to 0.6. The tip ECs probability of migration in the direction of the previous iteration (persistency) was left at 0.4, since there is no experimental evidence of increased persistency in the direction of growth after loss of ECs mechano-response.

To clarify this, we have added the following paragraph at lines 295-298:

"The tip ECs migration probabilities P1 and P2 were adjusted to account for the reduced organization of vessel structures observed experimentally upon inhibition of cell mechano-responders, such as ROCK, under cyclic stretch (26): P1 (persistency) was kept at 0.4, while P2 (randomness) was raised to 0.6."

15. On lines 301-302, the shape of a line differs from a straight line" perhaps refers to the shape of a vessel segment. Could the authors revise this?

Answer: thank you, we have removed the tortuosity analysis in the updated version of the manuscript since it was not enough sensitive to capture the vessel morphometric differences in the baseline model as compared to the mechano-response KO case. To show the increased shape defects after the KO of the ECs mechano-response, we have quantified the distribution of vessel lengths and amount of self-

intersecting vessels that generate closed loops and hence stop elongating. As a results, the mentioned sentence is not part of the manuscript anymore.

16. Line 320 states \immediately after surgery" but the figures show vessel distribution on the 7th day after the intervention.

Answer: thank you, it was an error. Strains were computed on the 7$^{th}$ day after surgery indeed. We have changed it in the manuscript.

17. I would like to suggest making the colour bar in Fig. 3A longer and indicating the key strain values for the assumptions of the model (5%, 10%, and 30%).

Answer: thank you for the hint, we added the strain values to the colour bar.

18. It might be helpful to add the strain distribution for the unloaded scenario with traction forces coming from cell activity only (to compare it to Fig. 3A).

Answer: thank you for the suggestion. We have added the strains distribution and directions of principal strains to Figure 8 and a description at lines 452-456:

"In the unloaded model, strains were induced by cell contractility only and their pattern reflect the OVSCs organization. As expected, absolute maximum principal strains within the healing region were reduced as compared to the loaded cases, below 2% (Fig. 8 A). Cellular traction forces induced mainly compressive strains within the callus, but tensile strains were predicted within the core of the gap (Fig. 8 A). Within the gap, principal strain directions were found to be aligned along the bone long-axis (Fig. 8 B). "

19. Could the authors clarify within the text how the orientation of stromal cells is related to the alignment of collagen fibers (e.g. around lines 370-372 and 477-479)? It is explained in more detail close to the end of Discussion, but for interpretation of the results, as they are reported, it would be helpful to have these details earlier.

Answer: thank you, we have provided more details, as follows (lines 397-401):

"Due to the lack of experimental data on OVSCs organization during the early stages of bone healing, OVSCs alignment was compared to the experimentally observed alignment of collagen fibres during the early healing phase (Fig. 5A). It has already been shown that ECM fibres align well with cellular orientation (20, 21). Indeed, cells are known to constantly remodel and align their ECM by exerting traction forces (27, 28) and, in turn, aligned ECM fibres influence cellular orientation and migration (29). "

**Reviewer #3**: This manuscript is focused on analyzing the effects of external and internal mechanical cues on neo-vessel growth and orientation. For that purpose, a femoral defect was introduced at each mouse's left femur, and after one week, the mice were euthanized, and different sections at the defect region were collected to observe the angiogenesis pattern. In addition, finite element and agent-based models of the femur defect were created to compare the computational analysis with the experimental outcome. Finally, in the computational model, the effects of the internal mechanical cues (sprout tip mechano-response and stromal cell traction force) and external loading were analyzed by inhibiting each in three different settings.

Answer: we thank the reviewer for attentively reading the manuscript.

The models appear to be seeded with random numbers. Is there variation in model results with repeated runs? If so, the authors should quantify and report the variance in their predictions. This would also provide them with the opportunity to perform statistical comparisons with their experimental data, which is currently lacking.

Answer: we thank the reviewer for the comment. We have provided a detailed answer to a similar point raised by reviewer #2 (the last part of question number 3). Briefly, in the updated version of the manuscript we have run 6 realizations for each model or scenario. All *ex vivo* and *in silico* results are presented as mean with standard deviation as a dispersion measure.
Following the reviewer's suggestion, we have performed a statistical comparison through a two-tail student t-test between *in silico* and *ex vivo* results for the vessel length density ratio. Results are included in Fig. 3 F-G. Concerning the distribution of vessel orientations, we have added a statistical analysis of *in silico* results via the Mann-Whitney U-test due to non-normality of the distribution. We did not statistically compare *in silico* and *ex vivo* results in this case because the bar plots in Fig. 4B already visually show that the *in silico* results do not match exactly the experiment. However, our objective was to capture the significant preferential alignment observed experimentally across different ROIs within two different fixation systems. We have included the normalized curves of the distribution of vessel orientations *in silico* vs. *ex vivo* in the Supporting Information file (S6 Fig.) to better compare their trends.

We have added and rephrased some sentences in the Results paragraph to clarify that the model could capture the preferential alignment of vessel sprouts:

Lines 370-371:

"Besides vessel density, *in silico* predictions appeared to replicate the preferential alignment of newly formed sprouts observed experimentally across the different ROIs (Fig. 4)."

Lines 379-384:

"Although *in silico* results did not match exactly the experimental ones in terms of average percentage of vessel structures aligned in a particular direction, the model could capture the significant preferential alignment observed experimentally. By comparing the *in silico* vs. *ex vivo* normalized curves of the distribution of vessel orientations across the ROIs (Supporting Information, S6 Fig.) it appears clear that the preferential alignment compares well, apart from the periosteal site (ROI 3) in the semirigid fixator case."

Why was only one histological image compared to model predictions? How much variation was there in the appearance of the histological images between animals?

Answer: thank you for the question. Although only one histological image is shown in our manuscript, we compared in total four *ex vivo* histological images per condition (rigid/semirigid) to our model predictions. Results of the vessel density analysis are reported for the individual samples in the scatter plot, while orientation results are reported as average ± standard deviation. The histological images look all alike.

The authors claim to have similar results from in-silico and ex-vivo setups. However, throughout the manuscript, it is evident that the computational analysis could not predict the experimental results based on vessel growth direction and density for the semirigid fixator group. It raises questions regarding the fidelity of the in-silico model. The lack of agreement in specific cases is likely due to the influence of other queues such as cytokine gradients and ECM orientation. This issue needs to be addressed in places where disagreement was reported.

Answer: thank you for the comment. We have added some sentences in the Results section where the lack of agreement in terms of vessel density and orientation under semirigid fixator is firstly reported:

Lines 358-360:
"However, under semirigid fixation, the model overestimated ($P$ value $< 0.05$) vessel density in ROI 2 (bone marrow). This discrepancy can be due to the constant rate of growth assigned to vessels that simplifies the complexity of the biological system."

Lines 377-385:
"However, periosteally (ROI 3), vessels were predicted to take a preferential horizontal orientation due to the high strains measured locally (above 20%), which was not observed experimentally (Fig. 4). Although *in silico* results did not match exactly the experimental ones in terms of average percentage of vessel structures aligned in a particular direction, the model could capture the significant preferential alignment observed experimentally. By comparing the *in silico* vs. *ex vivo* normalized curves of the distribution of vessel orientations across the ROIs (Supporting Information, S6 Fig.) it appears clear that the preferential alignment compares well, apart from the periosteal site (ROI 3) in the semirigid fixator case. This discrepancy is likely due to the influence of other factors, such as the haptotactic cues provided by ECM fibres, neglected in the model."

Moreover, we have further mentioned and discussed the lack of agreement in the Discussion section:

Lines 521-533:
"In the semirigid fixator scenario, the model predicted a preferential alignment of vessels periosteally in the direction perpendicular to the bone long axis as a consequence of the high strains predicted in this region, which was not observed experimentally. Other factors such as haptotactic cues (eg. ECM fibres), not included in the model and known to guide and direct vessel growth, might contribute to the vessel alignment observed in the *ex vivo* images. Indeed, the interaction of cells with matrix topology via contact guidance has been already proven to override the cell re-orientation in response to strains (30, 31). Interestingly, we show here that matrix fibres align parallel to the cortices along the bone periosteum, in line with the neo-vessel preferential alignment observed experimentally in the same region under both fixators. The contact guidance provided by this specific matrix organization at the periosteal site might prevent vessels from re-orienting as a response to high strains. By feeding the model with an experimentally measured fibres distribution and by including contact guidance, *in silico* prediction could better capture vessel patterning at the periosteal site under semirigid fixation, while results would not change under rigid fixation. "

Lines 597-602:
"We assumed a constant growth rate for vessels based on *in vivo* measurements (32), although it is known that ECs migration and proliferation rates are influenced by the externally applied load (33) and matrix stiffness (34). Future work should incorporate a mechanics-dependent vessel growth rate in the computer model, based on the available literature. This could help explain the reduced vascularity observed experimentally under semirigid fixation that the model failed to reproduce within the bone marrow (ROI 2). "

The finite element model did not completely reflect the experimental setup. For example, the authors used a finite element model with a fracture gap (0.5 mm) different from the experimental subject (0.7 mm). Although they mentioned a gap of 0.5 mm and 0.7 mm, the results would have been more acceptable if the FE model were similar.
The authors could explain why the model could not have a defect similar to the experimental one.

Answer: thank you for the comment. To address this concern, we adapted the model to replicate a 0.7 mm fracture gap and performed all the analyses again. All the results in this updated version of the manuscript are from the model with a 0.7 mm gap.

Details regarding the in-silico model are missing in the manuscript. This is crucial for reproducing and validating the results. For example, very little information is given regarding the agent-based model and its coupling with the finite element model.

Answer: we thank the reviewer for the comment. We have added more details regarding the coupling between the agent-based model and the finite element model (lines 155-162):

"After the initialization step, that represents the OVSCs and ECs seeding within the ABM, at every iteration, cellular traction forces are assigned to OVSCs and tip ECs based on their current position

and orientation within the healing region and are given as input to the FEM in the form of concentrated loads.  The finite element analysis is executed automatically, and the predicted principal strains and local deformations are read by the ABM. According to a set of rules describing the mechano-regulation of OVSCs and ECs organization that are detailed in the following paragraphs, OVSCs and ECs position and orientation are updated within the ABM and the algorithm starts over (Fig. 1). "

Further details about the agent-based model can be found in the paragraphs "Modelling sprouting angiogenesis" and "Modelling stromal cells activity". We have added a sentence to clarify this (lines 202-203):

„The specific mechano-biological rules guiding OVSCs organization and vessel growth are described in the following paragraphs."

Specific Comments:
Introduction
Page 4: "…external load that is actually transferred to the ECM within the healing region depends on the patient's physical activity but also on the gap size and the mechanical…" – This sentence is difficult to read. Consider rewriting with better clarity.

Answer: thank you for the suggestion. We rewrote the sentence to improve clarity (lines 92-94):

"In the clinical scenario of bone healing, mechanical strains within the ECM are determined not only by the patient's physical activity, but also by the gap size and the mechanical stability of the fracture fixation system."

Page 4: "it remains so far unknown how these different and dynamic mechanical signals impact cell self-organization" – In the same paragraph, it is mentioned how and why stromal cells and neo-vessels reorient due to mechanical signals. So, 'unknown' might be a strong word.

Answer: thank you for the comment. Although some studies provided insights on how and why stromal cells and neo-vessels reorient due to mechanical signals, it remains unclear in the specific context of early bone healing where dynamic mechanical signals act at different scales. We have rephrased the sentence to make it clearer (lines 94-96):

„In the specific context of early bone healing, however, it remains so far unknown how these dynamic mechanical signals acting at different scales impact cell self-organization and sprout patterning."

Page 4, Line 87: "… loads-induced…" should be …load-induced…".

Answer: thank you, we have changed it.

Page 5, Lines 110-111: Suggest citing original papers rather than review (37). This appears to be the first simulation study that included realistic microvessel traction forces: https://pubmed.ncbi.nlm.nih.gov/25429840/

Answer: thank you for the recommendation, we have modified the citation.

Materials and Methods

Page 6: "…on the left femur stabilized by a rigid or a semirigid external fixator (MouseExFix 100% and 50%, RISystem)." – The Authors should mention the actual material properties and other parameters used to model the fixator. This will help the reader to better understand the impact of using different fixators.

Answer: we thank the reviewer for the comment. We have added images of the 3D computer model in the Supporting Information (S2 Fig.) including fixators material properties. In addition, we have added a schematic illustrating the two fixators used experimentally to the Supporting Information (S1 Fig.).

Page 7: "However, collagen fibres have been reported to relate well with cell orientation" – The authors should provide stronger argument supporting their assumption that the orientation of collagen fibers can be used to identify the orientation of the stromal cells, or identify it as a limitation in the Discussion.

Answer: We thank the reviewer for the comment. We have added more arguments based on the literature to support our assumption (lines 166-172):

"OVSCs organization on day 7 was analysed in terms of cell orientations and compared to available experimental observations of collagen organization during the early stages of bone healing. In fact, to our knowledge, there are no experimental studies that have quantified OVSCs organization during the early stages of bone healing. However, collagen fibres have been reported to relate well with stromal cell orientation (20, 21). On the one hand, cells were shown to remodel and align the surrounding ECM by exerting traction forces (27, 28). On the other hand, aligned ECM fibres guide cellular orientation and migration (29).“

Page 8: "The models were meshed through a regular grid of 4 nodes plane strain elements…" – The authors have not mentioned anything about mesh sensitivity study. This study is needed to ensure the outcomes from computational analysis are not dependent on the size and shape of the mesh elements.

The authors also need to discuss the relationship between FE mesh resolution and ABM predictions. The FE mesh was used to define the ABM grid.

Answer: we thank the reviewer for the comment. A mesh sensitivity analysis was already performed on the original 3D finite element model. The optimal average characteristic element length size within the callus domain was 0.1 mm, which is bigger than the one chosen in this study (0.01 mm). Therefore, a mesh sensitivity study was not necessary in this particular case. We have added this detail to the Materials and Methods section (lines 192-194):

"A mesh convergence study was conducted for the 3D finite element model and resulted in an optimal average characteristic element size of 0.1 mm, one order of magnitude larger than the one adopted in the 2D model. "

In this study, we used the same grid size for the FE and the AB models; this facilitated the transfer of information between both models (mechanical stimuli to the ABM, cell traction forces to the FE model). The grid size was based on neo-vessel diameter reported in the literature during the first week of sprouting angiogenesis (35), where most vessels had a diameter below 15 µm. We tried to make it clearer in the text (lines 203-205, see below).

"Assuming an average neo-vessel and OVSCs diameter of 0.01 mm (35), coordinates of finite element nodes were used to create the 2D lattice grid of potential positions for cells. Therefore, the distance between lattice points was equal to 0.01 mm."

Page 8: "Displacement boundary conditions (Ux, Uy, Fig. 1) were applied to the cortical bone fragments which were derived from a previously developed and validated 3D finite element model of the same mouse osteotomy subjected to external physiological loading" – Beside mentioning that the boundary conditions were adopted from a previous paper, the authors should include more details in the manuscript for better understanding and flow of reading.

Answer: thank you for the suggestion. We have provided more details within the main text (lines 187-192, see below). Moreover, we have included an image in the Supporting Information file with dimensions and materials of the 3D finite element model (S2 Fig.).

“Specifically, a previously developed and validated 3D finite element model of a 0.5 mm mouse osteotomy stabilized with the same rigid and semirigid fixators and subjected to external physiological loading (7) was adapted to replicate the 0.7 mm defect induced in the current study (Supporting Information, S2 Figure). The displacement profiles of the cortices were recorded along the two orthogonal axis of the mid-longitudinal plane and applied as displacement boundary conditions (Ux, Uy, Fig. 1) to the cortical bone fragments of the 2D model.”

Page 9: Table 1 – The authors have not mentioned which factors were considered while estimating the values of these parameters. Clarifying this will help investigators in future research works and to reproduce the results.

Answer: thank you for your comment. All the parameters were taken from the available literature. This was not possible for the probabilities P1, P2 and P3, which were estimated through a parameter sweep analysis. We have provided additional information within the text (lines 224-227, see below) and more details can be found in the Supporting Information file (S4 file).

“To estimate the values of these probabilities, they were changed systematically through a parameter sweep analysis. The combination of values allowing to better match experimental data in terms of sprout patterning was selected and is reported in Table I. More details on the parameter sweep analysis are provided in the Supporting Information (S4 File). “

Page 9, Table 1: The authors should justify or discuss the use of a constant growth rate.

Answer: thank you for your comment. We have added a paragraph in the Discussion section, among limitations (lines 597-602):

“We assumed a constant growth rate for vessels based on *in vivo* measurements (32), although it is known that ECs migration and proliferation rates are influenced by the externally applied load (33) and matrix stiffness (34). Future work should incorporate a mechanics-dependent vessel growth rate in the computer model, based on the available literature. This could help explain the reduced vascularity observed experimentally under semirigid fixation that the model failed to reproduce within the bone marrow (ROI 2).”

Page 9, Table 1: P1 = P2 = 0.3, was this assumption investigated parametrically?

Answer: thank you for your comment, see two answers above.

Page 12: "Specifically, every 15 iterations, (to include a persistence time in a plane (34), all the potential positions surrounding…" – The use of parentheses and commas is confusing. The authors should revise this sentence.

Answer: thank you, we have revised the sentence and removed parentheses.

Page 14: "To assess this, we performed in silico experiments where tip ECs did not respond to the local mechanical signals, i.e. P3 was set equal to 0" – The authors did not mention the values of P1 and P2 in this scenario – 0.5?

Answer: thank you for the question. We have provided the answer in Question 14 from Reviewer #2 and clarified within the text.

Page 15: "Specifically, the number of vessels with a high T (T>1.2) and low T (T<=1.2) was compared" – The authors did not explain why the threshold value of 1.2 was chosen.

Answer: thank you for the comment. We have removed the tortuosity analysis in the updated version of the manuscript since it was not enough sensitive to capture the vessel morphometric differences in

the baseline model as compared to the mechano-response KO case. To show the increased shape defects after the KO of the ECs mechano-response, we have quantified the distribution of vessel lengths and amount of self-intersecting vessels that generate closed loops and hence stop elongating (lines 326-328).

"Vessel morphometric differences between the baseline and the EC_MR_KO models were quantified by computing the distribution of vessel lengths and the number of self-intersecting vessels that generate closed loops."

Page 16: "…under semirigid fixation, the model slightly overestimated vessel density…" – The authors should provide an explanation for why the model underestimated the vessel density at one region and overestimated at other region.

Answer: thank you for the comment. After averaging results over 6 realizations for each simulation, in silico results overestimate the vessel density observed experimentally within ROI2 (bone marrow) only, under semirigid fixation. Indeed, for every other ROI, in silico predictions and ex vivo results are not significantly different ($p>0.05$).
We have added an explanation either in the Results or in the Discussion section.

Lines 359-360:

"This discrepancy can be due to the constant rate of growth assigned to vessels that simplifies the complexity of the biological system."

Lines 597-602:

"We assumed a constant growth rate for vessels based on *in vivo* measurements (32), although it is known that ECs migration and proliferation rates are influenced by the externally applied load (33) and matrix stiffness (34). Future work should incorporate a mechanics-dependent vessel growth rate in the computer model, based on the available literature. This could help explain the reduced vascularity observed experimentally under semirigid fixation that the model failed to reproduce within the bone marrow (ROI 2)."

Page 17: Figure 3B – The direction of the strain direction is not clear from the image. The authors should include a color-coded reference to identify the direction from the color.

Answer: thank you for your input, we agree the principal strain directions were not clear. We have modified the figure by including a color-coded reference.

Page 19: "In silico predictions appeared to match with ex vivo observations in terms of sprouts alignment." – Do the predictions actually match? Although the trend of the bar chart is similar for ex vivo and in silico cases (Figure 4B and 4C), the change in orientation of vessels depending on strain is more prominent for in silico case. The vessel orientation is more random for ex vivo case. The random orientation is also evident in supplemental figure S4, where ROI 2 has almost similar distribution for all 20o bins for both rigid and semirigid cases. In addition, the orientation is opposite in ROI 3 for the semirigid fixator, which the authors failed to explain in their manuscript.

Answer: thank you for your considerations. We agree that the model could only capture the preferential alignment and the term "match" is not accurate. We have rephrased the sentence as follows (lines: 370-371):

" *in silico* predictions appeared to replicate the preferential alignment of newly formed sprouts observed experimentally across the different ROIs (Fig. 4)."

In addition, we have added (lines 379-384):

“Although *in silico* results did not match exactly the experimental ones in terms of average percentage of vessel structures aligned in a particular direction, the model could capture the significant preferential alignment observed experimentally. By comparing the *in silico* vs. *ex vivo* normalized curves of the distribution of vessel orientations across the ROIs (Supporting Information, S6 Fig.) it appears clear that the preferential alignment compares well, apart from the periosteal site (ROI 3) in the semirigid fixator case. “

We elaborated on the discrepancy between in silico and ex vivo results in ROI 3 for the semirigid fixator in the Discussion section (lines 521-533):

“In the semirigid fixator scenario, the model predicted a preferential alignment of vessels periosteally in the direction perpendicular to the bone long axis as a consequence of the high strains predicted in this region, which was not observed experimentally. Other factors such as haptotactic cues (eg. ECM fibres), not included in the model and known to guide and direct vessel growth, might contribute to the vessel alignment observed in the *ex vivo* images. Indeed, the interaction of cells with matrix topology via contact guidance has been already proven to override the cell re-orientation in response to strains (30, 31). Interestingly, we show here that matrix fibres align parallel to the cortices along the bone periosteum, in line with the neo-vessel preferential alignment observed experimentally in the same region under both fixators. The contact guidance provided by this specific matrix organization at the periosteal site might prevent vessels from re-orienting as a response to high strains. By feeding the model with an experimentally measured fibres distribution and by including contact guidance, *in silico* prediction could better capture vessel patterning at the periosteal site under semirigid fixation, while results would not change under rigid fixation. “

We have now added a sentence in the Results section, where this discrepancy is actually shown (lines 384-385):

“This discrepancy is likely due to the influence of other factors, such as the haptotactic cues provided by ECM fibres, neglected in the model.”

Although ROI 2 appears to have almost similar distribution for all 20° bins for both rigid and semirigid cases, the Mann-Whitney U-test with Bonferroni correction shows that differences are significant.

Page 22: "…no remarkable preferential orientation was observed when tip EC mechano-response was removed…" – Although the authors claimed that there was no preferred direction, Figure 6C shows that the percentage of vessels oriented in the 80o – 100o direction higher than all other directions. The authors should also explain the change in preferred direction of the vessels from the baseline case (0o - 20o and 160o-180o).

Answer: we thank the reviewer for the comment. With the new combination of probability values (P1,P2,P3) guiding tip ECs migration and after performing multiple realizations for each simulation, the KO of tip EC mechano-response led to a more clearly visible randomness in the distribution of vessel orientations (Fig. 6 A right). To confirm this observation, a statistical comparison was performed between the angle bins in which a higher percentage of vessel elements was detected (0°-20°,40°-60°,80°-100°,120°-140°,160°-180°). Due to the non-normality of certain datasets, the Kruskal-Wallis H test was carried out to compare the 5 angle groups. After assessing that there was a significant difference between groups, the post-hoc Mann Whitney U test, complemented by a Bonferroni correction, indicated that only the following pairs were significantly different: 80°-100°,120°-140° and 80°-100°,160°-180°.
We have added a description of the statistical comparison at lines 334-338 of the Materials and Methods section:

“Due to the non-normal distribution of certain dataset, the Kruskal-Wallis H test was carried out to compare n>2 groups. In case a significant difference between groups was detected, the post-hoc non-

parametric Mann-Whitney U-test was performed in Matlab (R2020b) for pairwise comparisons complemented by Bonferroni correction. The level of significance was defined as $p \leq 0.05$.”

We have also added a description of the output of the statistical comparison in the Results paragraph, lines 420-423:

“To confirm this observation, the angle bins in which a higher percentage of vessel structures was detected (0°-20°,40°-60°,80°-100°,120°-140°,160°-180°) were compared statistically and only the following pairs were determined to be significantly different: 80°-100° compared to 120°-140° and 80°-100° compared to 160°-180°.”

Page 24: "Interestingly, after removing the high extrinsic mechanical signal, the resulting vessel network differed depending on the presence or absence of cell traction forces." – Figure 8C does not imply a significant difference between the two cases. In all 20o bins, the percentage of vessels are similar for baseline and OVSC_TF_KO models.

Answer: we thank the reviewer for the comment, We agree the bar plot alone does not imply a significant difference between the two cases. We have therefore compared the Unloading baseline and Unloading OVSC_TF_KO statistically through a Mann Whitney U-test whose results are now displayed on the bar plot.
In addition, to confirm the preferential orientation along the 80°-100° bin in the Unloading Baseline and the mainly random orientation in the Unloading OVSC_TF_KO, we performed a statistical comparison between the angle bins in which a higher percentage of vessel structures was detected (0°-20°,40°-60°,80°-100°,120°-140°,160°-180°). Due to the non-normality of certain datasets, the Kruskal-Wallis H test was carried out to compare the 5 angle groups. After assessing that there was a significant difference between groups, the post-hoc Mann Whitney U test, complemented by a Bonferroni correction, was used to determine the pairs that were significantly different (indicated by the lines in the bar plot). We have added the following paragraph at lines 459-465:

“For both models, the angle bins in which a higher percentage of vessel structures was detected (0°-20°,40°-60°,80°-100°,120°-140°,160°-180°) were compared statistically. In particular, in the unloaded baseline model (Fig. 8 C left), the percentage of vessel structures within the gap (ROI1) that took a preferential orientation in the bone long axis direction was significantly higher compared to every other bin (Fig. 8 D). In the unloaded OVSC_TF_KO model, vessels grew straighter (Fig. 8 C right) and without any preferential alignment except for a significantly lower percentage of vessels detected in the 120°-140° bin (Fig. 8 D). “

Discussion

Page 26: "…the model slightly overestimated the vessel density within the endosteal gap and periosteally under semirigid fixation" – The authors mention here that the model overpredicted the vessel density in endosteal gap. However, Figure 3G contradicts their statement, where the in-silico vessel density is lower than experimental observation.

Answer: we thank the reviewer for the comment, that was a mistake. Under semirigid fixation, after running 6 realizations of the model, in silico results are not statistically different compared to ex vivo results within the gap and periosteum, while the model significantly overestimates the vessel density within the bone marrow. We have adjusted the text (lines 509-510).

Page 26: "The predicted preferential alignment of vessels was in agreement with the ex vivo data within different regions of interest for both fixators" – This statement does not qualify for all the cases described in this manuscript. As mentioned above, the alignment of vessel for semi rigid fixator was not similar for in silico and ex vivo cases.

Answer: we thank the reviewer for the comment and we agree that the sentence was not accurate. We have rephrased as follows (lines 513-515):

"The predicted preferential alignment of vessels within the three regions of interest was mostly in agreement with the *ex vivo* data under both fixators, although the model failed to capture the preferential alignment observed periosteally (ROI 3) under semirigid fixation."

Page 27, Lines 477-485: Please rewrite these paragraphs so they are not just 1 or 2 sentences.

Answer: we thank the reviewer for the suggestion and we have changed the paragraphs.

Page 27: "Other factors such as haptotactic cues (eg. ECM fibres), not included in the model and known to guide vessel growth, might be responsible for the vessel alignment observed in the ex vivo images." – The authors should shed more light on this topic as the finite element model could replicate the vessel alignment for rigid fixator without the 'other' factors mentioned here. The differences between rigid and semirigid fixator cases should be made clear, which are causing these discrepancies.

Answer: we thank the reviewer for the comment, and we have elaborated more on this topic (lines 526-533):

"Indeed, the interaction of cells with matrix topology via contact guidance has been already proven to override the cell re-orientation in response to strains (30, 31). Interestingly, we show here that matrix fibres align parallel to the cortices along the bone periosteum, in line with the neo-vessel preferential alignment observed experimentally in the same region under both fixators. The contact guidance provided by this specific matrix organization at the periosteal site might prevent vessels from re-orienting as a response to high strains. By feeding the model with an experimentally measured fibres distribution and by including contact guidance, *in silico* prediction could better capture vessel patterning at the periosteal site under semirigid fixation, while results would not change under rigid fixation."


1. Jørgensen PH, Bak B, Andreassen TT. Mechanical properties and biochemical composition of rat cortical femur and tibia after long-term treatment with biosynthetic human growth hormone. Bone. 1991;12(5):353-9.
2. Hosokawa A, Otani T. Ultrasonic wave propagation in bovine cancellous bone. J Acoust Soc Am. 1997;101(1):558-62.
3. Hosokawa A, Otani T. Acoustic anisotropy in bovine cancellous bone. J Acoust Soc Am. 1998;103(5 Pt 1):2718-22.
4. Lacroix D, Prendergast PJ. A mechano-regulation model for tissue differentiation during fracture healing: analysis of gap size and loading. Journal of Biomechanics. 2002;35(9):1163-71.
5. Claes LE, Heigele CA, Neidlinger-Wilke C, Kaspar D, Seidl W, Margevicius KJ, et al. Effects of mechanical factors on the fracture healing process. Clin Orthop Relat Res. 1998(355 Suppl):S132-47.
6. Goodship AE, Kenwright J. The influence of induced micromovement upon the healing of experimental tibial fractures. J Bone Joint Surg Br. 1985;67(4):650-5.
7. Borgiani E, Figge C, Kruck B, Willie BM, Duda GN, Checa S. Age-Related Changes in the Mechanical Regulation of Bone Healing Are Explained by Altered Cellular Mechanoresponse. J Bone Miner Res. 2019;34(10):1923-37.
8. Checa S, Prendergast PJ, Duda GN. Inter-species investigation of the mechano-regulation of bone healing: comparison of secondary bone healing in sheep and rat. J Biomech. 2011;44(7):1237-45.
9. Perier-Metz C, Duda GN, Checa S. Mechano-Biological Computer Model of Scaffold-Supported Bone Regeneration: Effect of Bone Graft and Scaffold Structure on Large Bone Defect Tissue Patterning. Front Bioeng Biotechnol. 2020;8:585799.
10. Perier-Metz C, Cipitria A, Hutmacher DW, Duda GN, Checa S. An in silico model predicts the impact of scaffold design in large bone defect regeneration. Acta Biomaterialia. 2022;145:329-41.

11. Borgiani E, Duda GN, Willie B, Checa S. BONE HEALING IN MICE: DOES IT FOLLOW GENERIC MECHANO-REGULATION RULES? FACTA UNIVERSITATIS Series: Mechanical Engineering. 2015;13(3):217-27.
12. Borgiani E, Duda GN, Checa S. Multiscale Modeling of Bone Healing: Toward a Systems Biology Approach. Front Physiol. 2017;8:287.
13. Borgiani E, Duda GN, Willie BM, Checa S. Bone morphogenetic protein 2-induced cellular chemotaxis drives tissue patterning during critical-sized bone defect healing: an in silico study. Biomech Model Mechanobiol. 2021;20(4):1627-44.
14. Leong PL, Morgan EF. Measurement of fracture callus material properties via nanoindentation. Acta Biomaterialia. 2008;4(5):1569-75.
15. Rosenfeld D, Landau S, Shandalov Y, Raindel N, Freiman A, Shor E, et al. Morphogenesis of 3D vascular networks is regulated by tensile forces. Proc Natl Acad Sci U S A. 2016;113(12):3215-20.
16. Gaggioli C, Hooper S, Hidalgo-Carcedo C, Grosse R, Marshall JF, Harrington K, et al. Fibroblast-led collective invasion of carcinoma cells with differing roles for RhoGTPases in leading and following cells. Nat Cell Biol. 2007;9(12):1392-400.
17. Shineh G, Patel K, Mobaraki M, Tayebi L. Functional Approaches in Promoting Vascularization and Angiogenesis in Bone Critical-Sized Defects via Delivery of Cells, Growth Factors, Drugs, and Particles. Journal of Functional Biomaterials [Internet]. 2023; 14(2).
18. Smeeing DPJ, Houwert RM, Briet JP, Groenwold RHH, Lansink KWW, Leenen LPH, et al. Weight-bearing or non-weight-bearing after surgical treatment of ankle fractures: a multicenter randomized controlled trial. European Journal of Trauma and Emergency Surgery. 2020;46(1):121-30.
19. Consigliere P, Iliopoulos E, Ads T, Trompeter A. Early versus delayed weight bearing after surgical fixation of distal femur fractures: a non-randomized comparative study. Eur J Orthop Surg Traumatol. 2019;29(8):1789-94.
20. Klebe RJ, Caldwell H, Milam S. Cells Transmit Spatial Information by Orienting Collagen Fibers. Matrix. 1990;9(6):451-8.
21. Kostyuk O, Brown RA. Novel spectroscopic technique for in situ monitoring of collagen fibril alignment in gels. Biophys J. 2004;87(1):648-55.
22. Kempen DHR, Creemers LB, Alblas J, Lu L, Verbout AJ, Yaszemski MJ, et al. Growth Factor Interactions in Bone Regeneration. Tissue Engineering Part B: Reviews. 2010;16(6):551-66.
23. Kretschmer M, Rudiger D, Zahler S. Mechanical Aspects of Angiogenesis. Cancers (Basel). 2021;13(19).
24. Wong S, Guo WH, Wang YL. Fibroblasts probe substrate rigidity with filopodia extensions before occupying an area. Proc Natl Acad Sci U S A. 2014;111(48):17176-81.
25. Checa S, Rausch MK, Petersen A, Kuhl E, Duda GN. The emergence of extracellular matrix mechanics and cell traction forces as important regulators of cellular self-organization. Biomech Model Mechanobiol. 2015;14(1):1-13.
26. Wilkins JR, Pike DB, Gibson CC, Li L, Shiu YT. The interplay of cyclic stretch and vascular endothelial growth factor in regulating the initial steps for angiogenesis. Biotechnol Prog. 2015;31(1):248-57.
27. Piotrowski-Daspit AS, Nerger BA, Wolf AE, Sundaresan S, Nelson CM. Dynamics of Tissue-Induced Alignment of Fibrous Extracellular Matrix. Biophysical Journal. 2017;113(3):702-13.
28. Davidson CD, Wang WY, Zaimi I, Jayco DKP, Baker BM. Cell force-mediated matrix reorganization underlies multicellular network assembly. Sci Rep. 2019;9(1):12.
29. Wang WY, Pearson AT, Kutys ML, Choi CK, Wozniak MA, Baker BM, et al. Extracellular matrix alignment dictates the organization of focal adhesions and directs uniaxial cell migration. APL Bioeng. 2018;2(4):046107.
30. Wang JH, Grood ES. The strain magnitude and contact guidance determine orientation response of fibroblasts to cyclic substrate strains. Connect Tissue Res. 2000;41(1):29-36.
31. Krishnan L, Underwood CJ, Maas S, Ellis BJ, Kode TC, Hoying JB, et al. Effect of mechanical boundary conditions on orientation of angiogenic microvessels. Cardiovasc Res. 2008;78(2):324-32.
32. Kenyon BM, Voest EE, Chen CC, Flynn E, Folkman J, D'Amato RJ. A model of angiogenesis in the mouse cornea. Investigative Ophthalmology & Visual Science. 1996;37(8):1625-32.

33. Burke D, Kelly DJ. A mechanobiological model of endothelial cell migration and proliferation. Comput Methods Biomech Biomed Engin. 2016;19(1):74-83.
34. Bordeleau F, Mason BN, Lollis EM, Mazzola M, Zanotelli MR, Somasegar S, et al. Matrix stiffening promotes a tumor vasculature phenotype. Proceedings of the National Academy of Sciences. 2017;114(3):492-7.
35. Nunes SS, Greer KA, Stiening CM, Chen HYS, Kidd KR, Schwartz MA, et al. Implanted microvessels progress through distinct neovascularization phenotypes. Microvascular Research. 2010;79(1):10-20.
